# Identifying RNA splicing factors using *IFT* genes in *Chlamydomonas reinhardtii*

**DOI:** 10.1098/rsob.170211

**Published:** 2018-03-07

**Authors:** Huawen Lin, Zhengyan Zhang, Carlo Iomini, Susan K. Dutcher

**Affiliations:** 1 Department of Genetics, Washington University School of Medicine, 4523 Clayton Avenue, St Louis, MO 63110, USA; 2 Department of Ophthalmology, Mount Sinai School of Medicine, New York, NY, USA

**Keywords:** IFT, RNA splicing, NMD, whole-genome sequencing, *Chlamydomonas*

## Abstract

Intraflagellar transport moves proteins in and out of flagella/cilia and it is essential for the assembly of these organelles. Using whole-genome sequencing, we identified splice site mutations in two *IFT* genes, *IFT81* (*fla9*) and *IFT121* (*ift121-2*), which lead to flagellar assembly defects in the unicellular green alga *Chlamydomonas reinhardtii*. The splicing defects in these *ift* mutants are partially corrected by mutations in two conserved spliceosome proteins, DGR14 and FRA10. We identified a *dgr14* deletion mutant, which suppresses the 3′ splice site mutation in *IFT81*, and a frameshift mutant of *FRA10*, which suppresses the 5′ splice site mutation in *IFT121*. Surprisingly, we found *dgr14-1* and *fra10* mutations suppress both splice site mutations. We suggest these two proteins are involved in facilitating splice site recognition/interaction; in their absence some splice site mutations are tolerated. Nonsense mutations in *SMG1*, which is involved in nonsense-mediated decay, lead to accumulation of aberrant transcripts and partial restoration of flagellar assembly in the *ift* mutants. The high density of introns and the conservation of noncore splicing factors, together with the ease of scoring the *ift* mutant phenotype, make *Chlamydomonas* an attractive organism to identify new proteins involved in splicing through suppressor screening.

## 1. Introduction

Pre-messenger RNA splicing, which removes noncoding introns from nascent RNAs to produce functional mRNAs, is an important and precisely controlled process. Some introns (Groups I, II and III), which are found in bacteria, fungi and organelles, are self-spliced. Splicing of most introns found in eukaryotic nuclei is facilitated by the spliceosome, which is a dynamic complex that contains multiple uridine-rich small nuclear ribonucleoproteins (snRNPs) and proteins associated with these snRNPs [1,2]. The major spliceosome, which is composed of U1, U2, U4/U6 and U5 snRNPs and associated proteins, recognizes conserved nucleotide sequences at the 5′ splice donor site (GT), the 3′ splice acceptor site (AG) and the branch site. Mutations in these splice sites usually cause aberrant transcripts and it is estimated that splice site mutations cause approximately 15% of human genetic diseases [3]. The minor spliceosome, which is composed of U11, U12, U4_atac_, U6_atac_ and U5 and many of the associated proteins found in the major spliceosome, is responsible for the removal of only approximately 0.3% of introns. It recognizes different conserved nucleotide sequences at the 5′ (AT) and 3′ (AC) splice sites. A few human diseases are associated with defects in the minor spliceosome [4]. Biochemical studies identified over 200 proteins associated with the major spliceosome and they can be grouped into different spliceosomal complexes. Some proteins are found to have core functions in the spliceosome while others are considered peripheral, and are present at specific points in the splicing process and are postulated to have noncore functions [2]. In our study described here, we focus on two peripheral proteins, DGCR14 and FRA10AC1 [5].

The *DGCR14* (DiGeorge syndrome (DGS) critical region gene 14) gene is located in the minimal DGS critical region on human chromosome 22. DGS (velo-cardio-facial syndrome or 22q11 deletion syndrome) is caused by a deletion of about 46 genes within an approximately 2.5 Mb region and is associated with heart defects, cleft palate, low levels of calcium in the blood, poor immune system and delayed physical and social developments [6]. DGCR14 is a highly conserved protein [6–8] that localizes to the nucleus [7–9]. In *Schizosaccharomyces pombe*, deletion of the DGCR14 homologue Bis1 affects cell viability during stationary growth but not exponential growth [7]. In *Caenorhabditis elegans*, while loss of function of *ESS-2*/*DGCR14* in wild-type worms has no obvious defects, a loss of function *ess-2* allele in mutants with splice acceptor site mutations affects the stability of both correctly and incorrectly spliced transcripts. DGCR14/ESS-2 was proposed to facilitate splicing [8].

When cells are exposed to partial DNA replication stress, gaps, constrictions or breaks are likely to form at specific sites along the chromosome. Those are considered chromosomal fragile sites [10]. A rare group of chromosomal fragile sites are induced by exposure to folate, and the most frequent folate-sensitive human autosomal fragile site occurs at 10q23 [11]. A CCG expansion in the 5′ UTR of a gene, *FRA10AC1* (FRA10A associated CGG repeat 1), is proposed to create the fragile site. The conserved FRA10AC1 (C10orf4) protein [12] was identified as a spliceosomal protein [13,14] and it localizes to the nucleus [15]. Yeast two-hybrid assays revealed that FRA10AC1 interacts with DGCR14 [2]. No functional study of the involvement of FRA10AC1 in pre-mRNA splicing has been reported.

Pre-mRNA splicing defects can lead to accumulation of aberrant transcripts, which can be deleterious to cells [3]. Degradation of these aberrant transcripts, which usually harbour premature termination codon (PTC), is controlled by the nonsense-mediated mRNA decay (NMD) surveillance system. The NMD machinery contains three conserved core components, UPF1, UPF2 and UPF3, which are found in all eukaryotic cells [16]. Phosphorylation of the RNA helicase UPF1, usually performed by the kinase SMG1, regulates NMD in some eukaryotes. In mouse, *SMG1* is required for embryogenesis. About 9% of PTC-containing alternatively spliced transcripts show significant increase in *SMG1*-depleted mouse cells [17]. In *C. elegans*, transcripts with nonsense mutations accumulate in *smg1* mutants [18]. In *Drosophila*, a likely null *smg1* mutant has only a modest effect on NMD efficiency [19]. *SMG1* is present in other land plants but not in *Arabidopsis thaliana*. No *SMG1* gene has been identified in either *Saccharomyces cerevisiae* or *S. pombe* [20].

Intraflagellar transport (IFT) is a process that moves proteins between the cell body and the cilia/flagella, which are microtubule-based organelles that protrude from the cell body. This bidirectional process is essential for the formation and maintenance of the flagellum. The unicellular green alga *Chlamydomonas reinhardtii* assembles two flagella that confer the ability to swim in liquid medium. Mutations in *IFT* genes affect flagellar assembly and the mutant phenotypes are easily detectable due to the inability to oppose gravity by swimming [21–23].

In this study, we used whole-genome sequencing to identify splice site mutations in two IFT genes, *IFT81* and *IFT121*. The missplicing events of *IFT81* and *IFT121*, which include intron retention, exon skipping and adoption of new splice sites, can be corrected by mutations in either *DGCR14* or *FRA10AC1*, but not by mutations in *SMG1*. Our study provides the first functional study of the involvement of FRA10AC1 in pre-mRNA splicing and suggests that, like *C. elegans*, the *Chlamydomonas* DGR14 protein is involved in pre-mRNA splicing regulation. As in other organisms, the *Chlamydomonas* SMG1 protein is involved in NMD. These *ift* mutants provide a new resource to identify new players in RNA splicing through suppressor screening, and *Chlamydomonas* serves as a tractable model system to study RNA splicing.

## 2. Material and methods

### 2.1. Strains and culture conditions

Strains were obtained from the *Chlamydomonas* Resource Center at the University of Minnesota. They include *fla9*, CC-1918; LMJ.RY0402.144851; and S1D2, CC-2290. The *fla9* strain was backcrossed multiple times to wild-type cells to remove any unlinked modifiers. These strains were routinely maintained on Sager and Granick (R) medium agar plates. Ultraviolet mutagenesis to isolate the *ift121-2* mutant and to screen for suppressors was performed as previously described [24].

**Figure 1.**
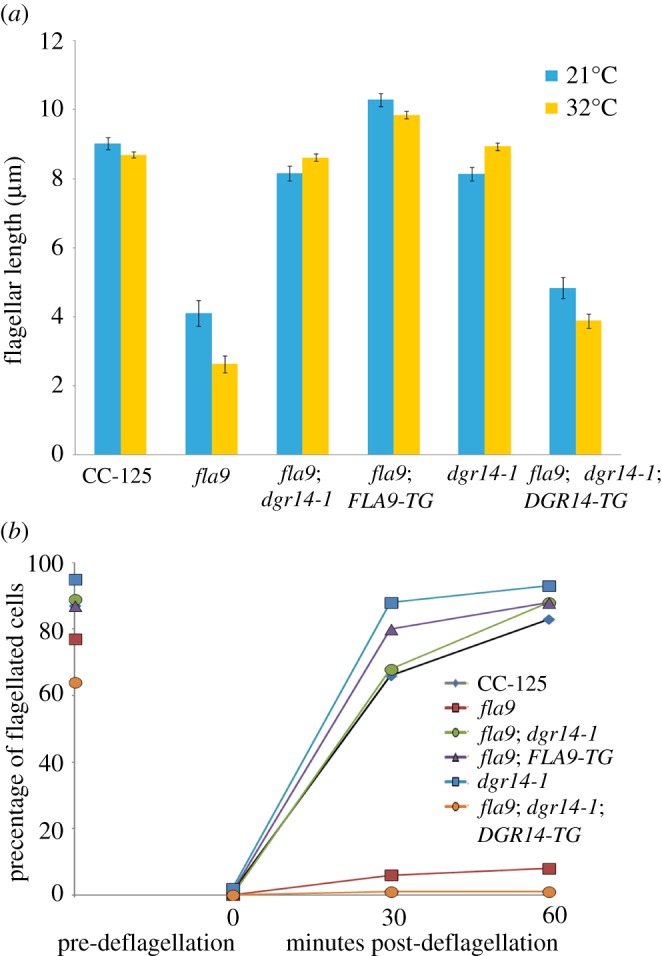
Flagellar assembly and regeneration defects in *fla9* can be rescued by both wild-type *IFT81* gene and a suppressor mutation, *dgr14-1*. (*a*) Measurement of flagellar length (*n* = 100) in individual strains at both 21°C (blue) and 32°C (yellow). Bars indicate the standard deviation of the mean. (*b*) Percentage of flagellated cells (*n* = 100) in each strain before and after flagellar amputation by pH shock. Cells were kept at 32°C during flagellar regeneration.

**Figure 2.**
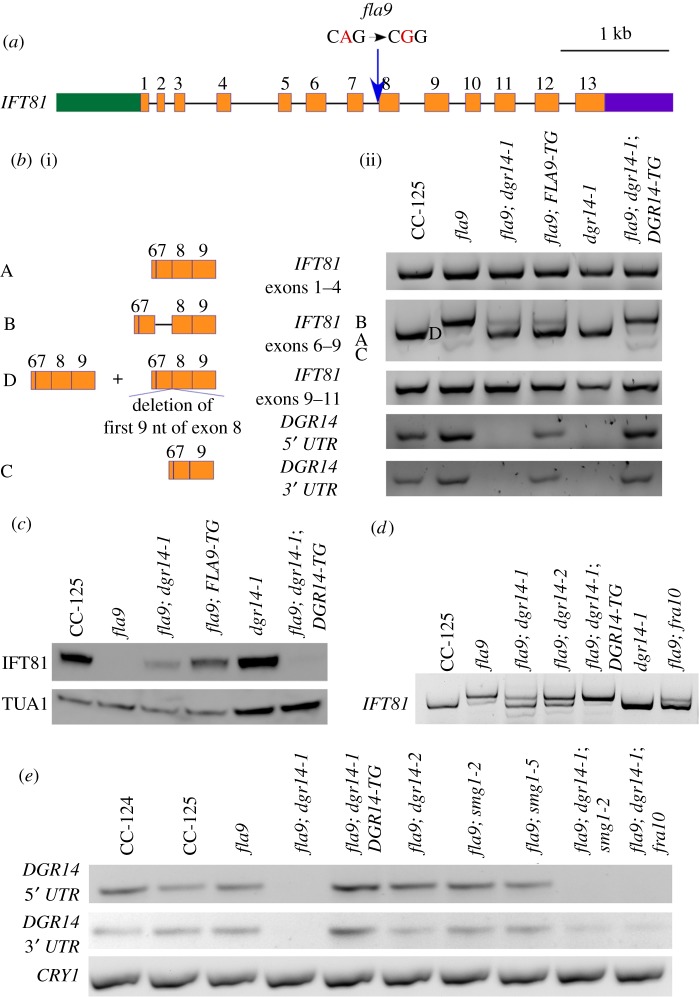
A splice acceptor site mutation affects splicing of *IFT81*. (*a*) Gene structure of *IFT81* and position of the *fla9* mutation. Green box, 5′ UTR; orange boxes, exons; black solid lines, introns; purple box, 3′ UTR; blue arrow, position of the *fla9* mutation. The single-nucleotide change from A to G is indicated in red. (*b*) Alternative splicing of *IFT81* in cells grown at 21°C. (i) Representation of multiple *IFT81* transcripts between exons 6 and 9. (ii) RT-PCR products of *IFT81* between exons 1–4 (top), exons 6–9 (middle), and exons 9–11 (bottom). Individual bands are labelled according to their compositions. (*c*) Immunoblot of IFT81. Ten micrograms of flagellar proteins isolated from cells grown at 21°C were used in each lane. The same membrane was later probed with an anti-α-tubulin (TUA1) antibody to serve as a loading control. (*d*) RT-PCR of *IFT81* exons 6–9 in various strains grown at 25°C. (*e*) RT-PCR of *DGR14* at both 5′ and 3′ ends of the gene. Amplification of the ribosomal protein gene *CRY1* serves as a loading control.

**Figure 3.**
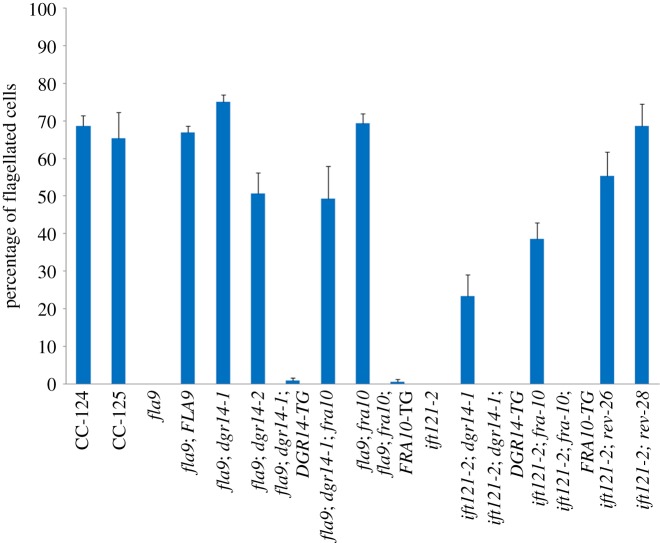
Flagellar assembly in splice site *ift* mutants is restored by mutations in splicing factors. The percentage of flagellated cells was determined by counting 100 cells in triplicates in each strain. Error bars represent the standard deviation of the mean.

The *fla9* cells, when first obtained from the *Chlamydomonas* Resource Center, displayed a temperature-sensitivity phenotype as reported previously [25]. These cells maintained their flagella and swimming ability at 21°C and became aflagellate when cells were shifted to 32°C. Thus, we were able to analyse flagellar phenotype from *fla9* cells, described in figures 1 and 2. Approximately 2 years after these initial studies, we noted the *fla9* cells become aflagellate at all temperatures tested (21°C, 25°C and 32°C). To exclude any putative spontaneous mutations, we performed at least five rounds of meiotic crosses and analysed over 300 progeny. The identified *IFT81* mutation in *fla9* always cosegregates with the aflagellate phenotype (figure 3) and the same splicing pattern of *IFT81* persists in the aflagellate cells (figure 2*d*). The *fla9; dgr14-1; DGR14-TG* cells, which had short flagella similar to *fla9* when first identified (figure 1*a*), become aflagellate during the same period (figure 3). Missplicing of *IFT81* remains the same in *fla9*; *dgr14-1*; *DGR14-TG* (figure 2*d*). In addition to the *fla9* strain we maintained in the laboratory, we acquired *fla9* from the *Chlamydomonas* Resource Center. We tested the flagellar phenotype of these two *fla9* strains, *fla9*; *dgr14-1*, and *fla9*; *dgr14-1*; *DGR14-TG* in both R and TAP media, with trace elements obtained from the *Chlamydomonas* Resource Center and a different *Chlamydomonas* laboratory, at 21°C. The aflagellate phenotype persists in both *fla9* strains and in *fla9*; *dgr14-1*; *DGR14-TG* while *fla9*; *dgr14-1* displays more than 80% flagellated cells in the same media. We used EDTA acid to prepare trace elements, which led to no precipitation in the final product, while other trace elements were prepared with sodium EDTA that resulted in precipitation and filtration to obtain the final product [26]. Therefore, difference in trace elements does not contribute to the change of the *fla9* phenotype. Given the phenotype observed, we consider the *fla9* mutant in our hands has lost its temperature-sensitive phenotype and the aflagellate phenotype at all temperatures is the *fla9* mutant phenotype we study onward.

### 2.2. Meiotic mapping of *fla9* and whole-genome sequencing

A cross between *fla9* and wild-type (CC-124) showed 2 : 2 segregation of the aflagellate phenotype at 32°C in 87 tetrads. It suggests that the *fla9* mutant contains either a single mutation or multiple tightly linked mutations. *fla9* was mated to a highly polymorphic strain S1D2 (CC-2290) [27] and in 235 meiotic progeny *fla9* maps between 3.255 and 3.780 Mb on chromosome 17 in Phytozome v. 5.5 of the *Chlamydomonas* genome. *Chlamydomonas* genomic DNA for whole-genome sequencing was prepared as previously described [24,28]. Three micrograms of DNA were submitted to the Genome Technology Access Core (Washington University) for library construction, Illumina sequencing and initial data analysis. SNP calling and subtraction of irrelevant SNPs/short indels were performed as previously described [28]. Large indels were identified by SoftSearch [29]. Around 12 000 breakpoints were found in strain 4c that was identified as an extragenic suppressor and compared to those found in the *pf27* strain [30] and in *fla11-2* [31] to identify indels unique to the 4c strain.

### 2.3. cDNA preparation, TA cloning and sequencing

For RNA isolation, cells from two R medium agar plates grown for 5 days were resuspended in 40 ml nitrogen-free medium (M-N/5) for 2 h at room temperature to allow flagellar assembly. The cells were then collected and RNA extraction was performed with the RNeasy Mini Kit (Qiagen) according to the manufacturer's recommendation. Two micrograms of RNA was used in a reverse transcription reaction with SuperScript III (Invitrogen) with random primers as previously described [32]. Gel-purified reversed transcribed cDNA products generated by Phusion (New England Biolabs) at the annealing temperature of 64°C were either subjected to direct Sanger sequencing (GeneWiz) or subjected to TA cloning. Primer sequences used in this study are listed in the electronic supplementary material, table S1. For TA-cloning, poly(A) tails were added to the gel-purified PCR products with TAQ polymerase and the fragments were later cloned into the pCR4-TOPO vector (Invitrogen). Plasmid DNA for Sanger sequencing was prepared by FastPlasmid Mini Kit (5 Prime) and sequenced with both T3 and T7 primers at GeneWiz.

### 2.4. BAC DNA preparation and *Chlamydomonas* transformation

*Chlamydomonas* BAC DNA was prepared using a QIAGEN Midiprep kit as previously described [33]. For rescue of *fla9*, two micrograms of isolated BAC DNA was transformed into *fla9* cells by electroporation [32]. Cells were separated into 96 tubes each containing 20 ml liquid rich medium at 32°C. Swimming cells in these tubes were enriched by transferring the top 5 ml liquid into fresh 20 ml liquid rich medium every two days. After five rounds of transfer, crude DNA preparation, PCR and enzyme digestion to identify both mutant and transformed *IFT81* genes were performed from all transformants. For rescue of 4c, the 40B10 BAC DNA was digested with *Hin*dIII and *Sbf*I to obtain a 7.5 kb fragment, which includes approximately 3.5 kb upstream of the start codon of *DGR14*. The fragment was then cloned into *Hin*dIII and *Pst*I sites of a pBlueScript SK vector (Stratagene). For rescue of *fra10*, the 3.1 kb genomic DNA fragment was amplified by FRA10-1F and FRA10-1R (electronic supplementary material, table S1) using Phusion DNA polymerase followed by the addition of poly(A) with Taq DNA polymerase for 10 min at 72°C. The amplified fragment was then cloned into the pCR4-TOPO vector (Invitrogen).

### 2.5. Flagellar length measurement

To measure *Chlamydomonas* flagellar length, *Chlamydomonas* cells were resuspended in liquid M-N/5 medium for 4 h and treated with autolysin for 30 min at room temperature. Cells were then resuspended in microtubule stabilization buffer (MTSB) [34] at room temperature. Multi-well slides (ThermoFisher) were coated with 0.1% poly-l-lysine (Sigma-Aldrich) for 5 min before being washed with MilliQ water once and allowed to dry completely. Cells were applied to wells on the slides and left in the dark for 2 min at room temperature. Excess cell suspension was removed by pipetting. Lysis buffer (MTSB + 1% Nonidet P-40) was added to individual wells and cells were lysed for 2 min at room temperature. Excess cell suspension was removed by pipetting. To wash off the lysis buffer, MTSB was added to individual wells and removed by pipetting. Samples were fixed with MTSB + 4% paraformaldehyde for 30 min at room temperature. Slides were then submerged in cold methanol (−20°C) for 2 × 5 min and left to dry at room temperature. Nucleo-flagellar apparatuses [35] are attached to individual wells and are visible under a phase-contrast microscope. The samples were blocked by 100% blocking buffer (BB, 5% BSA and 1% fish gelatin in PBS) for 1 h at room temperature. The samples were stained with a primary antibody (anti-acetylated α-tubulin, Sigma) at 1 : 500 dilution with 20% BB at 4°C overnight. The samples were washed six times with 20% BB, followed by 1-h inoculation at room temperature with a secondary antibody (Alexa-594-conjugated goat anti-mouse, Invitrogen) at 1 : 1000 dilution with 20% BB. The samples were washed six times with 20% BB and mounted in Fluoromount-G (SouthernBiotech). The images were captured with an UltraVIEW VoX laser spinning disk confocal microscope (PerkinElmer) and acquired by Volocity software (PerkinElmer). ImageJ was used to measure 100 flagella from 50 cells from each strain.

### 2.6. Flagellar regeneration

Flagellar amputation of *Chlamydomonas* cells was performed by pH shock [36]. After pH neutralization, cells were pelleted by centrifugation (1000 × *g*, 2 min) and resuspended in fresh R medium. The cells were kept at 32°C and a small portion of cells was fixed in 0.2% glutaraldehyde at each time point for visualization and cell count.

### 2.7. Immunoblot

*Chlamydomonas* flagellar isolation was performed as previously described after dibucaine amputation [37]. Ten micrograms of flagellar proteins were used in each strain. Immunoblots were performed as previously described [38]. The primary antibodies used include IFT81.1 (a gift from Dr. Doug Cole, 1 : 350 dilution) and anti-α-tubulin (DM1A, Sigma-Aldrich, 1 : 5000 dilution). The secondary antibody used was HRP-conjugated goat anti-mouse antibody (Bio-Rad, 1 : 5000 dilution).

### 2.8. Protein sequence alignment and prediction of protein structures

Protein sequences were obtained from NCBI and they were aligned by MUSCLE [39]. Colour-coded alignment of protein sequences was obtained using COLORFY [32]. Coiled-coil domains were predicted using COILS [40] and α-helices were predicted by YASPIN [41].

### 2.9. Analysis of orthologues of DGR14 and FRA10 in eukaryotes

Orthologues of DGR14 and FRA10 were obtained from the EggNog database [42]. To ensure that absence of an orthologue is not due to incomplete genome assembly, we required species used in the analysis to contain at least four out of five core splicing proteins, U2AF, PRP8, PRP17, PRP19 and SLU7. The absence of an orthologue in each species is also verified by BLAST against proteins in the NCBI database. Intron density was acquired from Rogozin *et al.* [43] and a median density is reported when there are multiple species in a given class.

## 3. Results

### 3.1. The *fla9* mutant contains a splice site mutation in *IFT81*

The *fla9* mutant was isolated as a temperature-sensitive mutant in an *N*-methyl-nitro *N*-nitrosoguanidine mutagenesis screen [25]. The cells grow flagella at 21°C and maintain their flagella at 32°C for 6 h. However, the cells fail to regenerate flagella at 32°C and thus become aflagellate once they go through cell division at 32°C. When we first obtained the *fla9* strain from the *Chlamydomonas* Resource Center, they displayed the mutant phenotype as expected (figure 1). The *fla9* cells had short flagella (approx. 4 µm) when compared to wild-type (CC-125) cells (approx. 9 µm) at 21°C. While the wild-type cells maintained their flagellar length 4 h after they were switched to 32°C, the *fla9* cells had even shorter flagella (approx. 2.6 µm) (figure 1*a*). Prolonged (overnight) inoculation at 32°C eventually resulted in aflagellate cells. Only approximately 5% of *fla9* cells were capable of regenerating flagella 1 h after pH shock [36] at 32°C, compared to approximately 80% of wild-type (CC-125) cells (figure 1*b*).

The *fla9* allele was previously mapped to chromosome 17 [44] and we mapped it to a region between 3.255 and 3.780 Mb. Whole-genome sequencing of *fla9* identified only one change (AG to GG) in the region (table 1) and it affects the 3′ splice site of intron 7 in the *IFT81* gene (figure 2*a*) [38]. We designed a PCR-based assay to detect this change (electronic supplementary material, table S1) and it cosegregated with the flagellar defect in 50 meiotic *fla9* progeny. Thus, it is tightly linked to the *fla9* mutant.

**Table 1.** Summary of mutants identified in this study.

mutant	affected gene	mutation	position
*fla9*	*IFT81*	c. 823-2A > G	chromosome_17: 3 365 104
*dgr14-1*	*FAP208*, *FAL13*, *Cre11.g482101*, *Cre11.g482150*, *FBB9*, *Cre11.g482250*	33 kb deletion	chromosome_11: 3 603 615–3 636 297
*ift121-2*	*IFT121*	c. 2754 +1G > A	chromosome_11: 2 411 434
*ift121-2 rev26*	*IFT121*	c.2748G > A, p. K916 K	chromosome_11: 2 411 441
*ift121-2 rev28*	*IFT121*	c. 2820 + 1G > A	chromosome_11: 2 411 221
*fra10*	*Cre07.g336250*	c. 600delC, p. K201fs	chromosome_7: 3 538 004
*smg1-1*	*Cre13.g572050*	c. 2263C > T, p. Q755X	chromosome_13: 1 413 437
*smg1-2*	*Cre13.g572050*	c. 5587G > T, p. E1863X	chromosome_13: 1 417 593
*smg1-3*	*Cre13.g572050*	c. 6787A > T, p. K2263X	chromosome_13: 1 419 322
*smg1-4*	*Cre13.g572050*	c. 14452G > T, p. E4818X	chromosome_13: 1 430 695
*smg1-5*	*Cre13.g572050*	c. 14494G > T, p. E4832X	chromosome_13: 1 430 737

We transformed 1E18 BAC (chromosome 17, 3 318 187–3 378 344) DNA into the *fla9* mutant [45,46]. Ninety-six independent transformants were recovered after enriching for cells that regain the ability to swim at 32°C. Eighty-one of them had both mutant and wild-type alleles based on the PCR-based assay. The remaining 15 transformants, which still have the mutant allele, may carry suppressor mutations occurring elsewhere, but were not studied further. Backcrosses of nine randomly selected rescued transformants showed the rescue event is extragenic. One of such rescued strain (*fla9*; *FLA9-TG*) was randomly selected for further analysis. This rescued strain has approximately 10 µm flagella at both 21°C and 32°C (figure 1*a*) and it regenerates flagella at 32°C, similar to what we observe in wild-type cells (figure 1*b*).

### 3.2. The splice site mutation in *fla9* leads to alternative splicing of *IFT81*

We expect the change at the 3′ splice site in *fla9* to affect splicing of the *IFT81* gene. PCR fragments from exons 1–4 and exons 9–11 are identical in length and intensity between wild-type and *fla9* strains (figure 2*b*), which shows that the stability of the *IFT81* mRNA is not affected in the *fla9* strain. By contrast, PCR fragments from exons 6–9 show differences in wild-type and *fla9* cells. In wild-type CC-125 cells, a single band, that corresponds to the predicted length of exons 6–9, is observed (figure 2*b*, band A). In *fla9* cells, three bands are amplified (figure 2*b*, bands B, D and C). Sanger sequencing indicates that the predominant band B includes intron 7, and that exon 8 is skipped in band C (figure 2*b*). The middle band (D) contains a mix of wild-type and misspliced products. This band was subcloned and nine single colonies were subjected to Sanger sequencing. Two different splicing products are found: four different single colonies contain the wild-type cDNA and the other five colonies have a deletion of the first nine nucleotides of exon 8. The deletion is predicted to remove three amino acids (V_275_N_276_E_277_) in IFT81 protein. The glutamic acid is conserved in all IFT81 proteins examined (electronic supplementary material, figure S1). Nevertheless, an immunoblot using anti-IFT81 monoclonal antibody [47] shows that the IFT81 protein is absent in the *fla9* mutant at both 21°C (figure 2*c*) and 32°C (electronic supplementary material, figure S2). In the *fla9* strain rescued with wild-type *FLA9* (*fla9*; *FLA9-TG*), the wild-type cDNA (band A) becomes the predominant species but the rescued strain still shows low levels of intron inclusion and exon skipping fragments (bands B and C) (figure 2*b* and electronic supplementary material, figure S2). Correspondingly, the IFT81 protein is expressed in the *fla9*; *FLA9-TG* strain at a lower level than found in wild-type cells (figure 2*c* and electronic supplementary material, figure S2).

### 3.3. Mutations in the *DGCR14* gene suppress the flagellar defect in *fla9*

A *fla9* strain with a spontaneous mutation (4c) shows flagellar assembly and regeneration at 32°C (figure 1, *fla9; dgr14-1*). By PCR and enzyme digestion, the *fla9* splice site mutation is still present in 4c. Similar to the *fla9*; *FLA9-TG* strain, the major RT-PCR band amplified by the *IFT81* exon 6–9 primers in the 4c strain is the wild-type product at both 21°C and 32°C (figure 2*b* and electronic supplementary material, figure S2). We detected the IFT81 protein in 4c cells (*fla9*; *dgr14-1*) but the protein abundance is lower than that found in the *fla9*; *FLA9-TG* strain at both temperatures (figure 2*c* and electronic supplementary material, figure S2).

A meiotic cross between 4c and the wild-type strain shows that 4c carries an extragenic mutation that is unlinked to the *fla9* mutation (*n* = 23). The suppressor mutant itself (*dgr14-1*) has no flagellar assembly or regeneration defect (figure 1). To identify the causative mutation in 4c, we subjected one of the suppressed meiotic progeny to whole-genome sequencing. With 157× coverage of the genome, we did not identify a causative SNP or short insertion/deletion (indel) (electronic supplementary material, table S2) [28]. Instead, we identified a 32 682-bp sequencing gap on chromosome 11: 3 603 615–3 636 297 by SoftSearch [29] and manual examination of aligned reads. Within this region, six genes, *FAP208*, *FAL13*, *Cre11.g482101*, *Cre11.g482150*, *FBB9* and *Cre11.g482250*, are either missing or disrupted. Both FAP208 and FBB9 were identified as flagellar proteins [48,49]. *Cre11.g482101*, *Cre11.g482150* and *Cre11.g482250* are novel genes. The *FAL13* gene contains five exons and encodes a protein of 699 amino acids and shares 30% protein sequence identity (3 × 10^−12^) with the human DGCR14 protein. Sequence alignment indicates that it shares sequence similarity to DGCR14 homologues in *S. pombe*, *Arabidopsis*, *Drosophila*, *C. elegans*, zebrafish, mouse and human (electronic supplementary material, figure S3). Owing to its sequence similarity and its putative function in mRNA splicing, we renamed the *FAL13* gene as *DGR14*.

Given that DGCR14 has been implicated in mRNA splicing in *C. elegans* and the splicing pattern of *IFT81* is altered in the 4c strain, we expect introduction of the wild-type *DGR14* gene into the *fla9*; *dgr14-1* double mutant leads to short flagella and missplicing of *IFT81* as observed in *fla9*. An approximately 7.5 kb DNA fragment, which contains the full-length *DGR14* gene and approximately 3.5 kb upstream of *DGR14* (part of *Cre11.g482101*), was transformed to the *fla9*; *dgr14-1* double mutant [50]. Four transformants with short or no flagella were identified. One of these transformants does not contain a wild-type *DGR14* gene. It suggests that the flagellar phenotype is likely due to a random insertion event instead of *DGR14* rescue, which is known to occur [51]. The other three transformants carry the transformed wild-type *DGR14* gene. We randomly picked one of these transformants for RT-PCR and Sanger sequencing of *IFT81* splicing products from exons 6 to 9 (figure 2*b*, *fla9; dgr14-1; DGR14-TG*). They are identical to those found in *fla9* (figure 2*b*). Similar to *fla9*, the *fla9*; *dgr14-1*; *DGR14-TG* cells have short flagella (approx. 4 µm) at both 21°C and 32°C (figure 1*a*) and they fail to regenerate flagella after amputation at 32°C (figure 1*b*). No IFT81 protein is detected in *fla9*; *dgr14-1*; *DGR14 -TG* cells (figure 2*c* and electronic supplementary material, figure S2). In contrast, the *dgr14-1* mutant has wild-type *IFT81* transcript (figure 2*b* and electronic supplementary material, figure S2) and wild-type IFT81 protein (figure 2*c* and electronic supplementary material, figure S2). Therefore, we conclude that the *dgr14-1* deletion suppresses the *fla9* mutant phenotype by modifying the misspliced *IFT81* transcripts.

In addition to *dgr14-1*, we obtained a *Chlamydomonas* strain LMJ.RY0402.211897, which has an insertion in intron 5 of *DGR14*, from the *Chlamydomonas* Library Project (CLiP) [52]. The insertion leads to reduced level of the *DGR14* transcript at the 3′ UTR (figure 2*e*). This insertional allele is renamed *dgr14-2*. It was crossed to the 4c strain and all 22 independent *fla9* progeny that contain the *dgr14-2* allele show normal flagellar assembly (figure 3, *fla9; dgr14-2*). Examination of *IFT81* exons 6–9 in one of these progeny indicates that while mRNA with intron retention is observed, the wild-type transcript is restored (figure 2*d*). Therefore, both a deletion (*dgr14-1*) and an insertion (*dgr14-2*) in *DGR14* act as suppressors of the *fla9* mutant.

### 3.4. A splice site mutation in *IFT121* causes alternative splicing of *IFT121*

**Figure 4.**
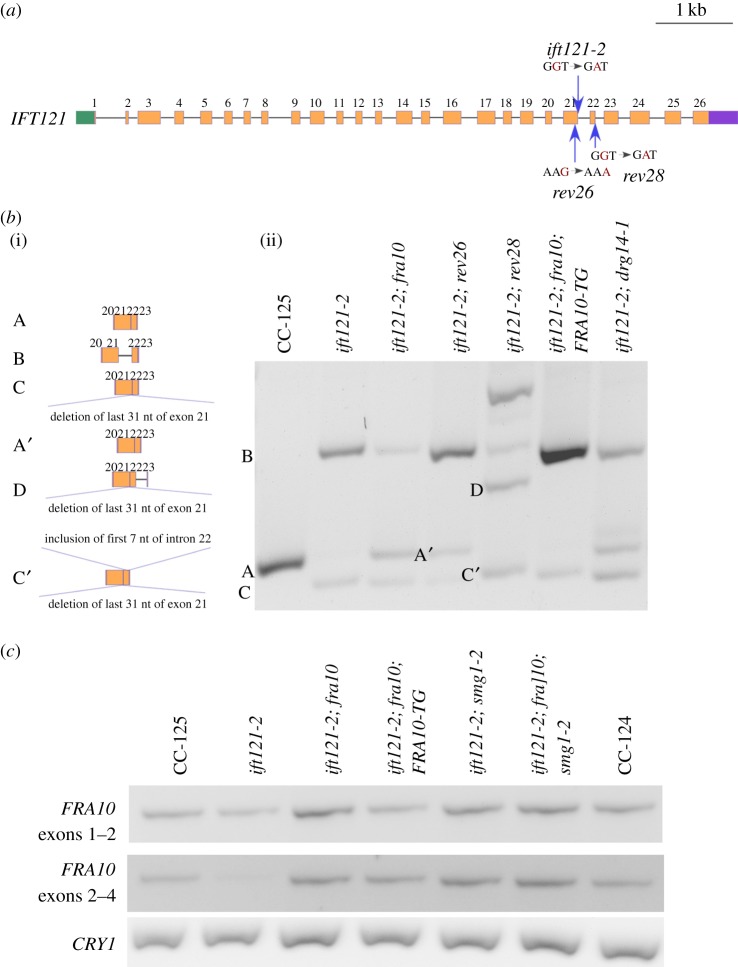
A splice donor site mutation affects splicing of *IFT121*. (*a*) Gene structure of *IFT121* and positions of multiple mutations. Green box, 5′ UTR; orange boxes, exons; black solid lines, introns; purple box, 3′ UTR; blue arrows, positions of *ift121* mutations. The single-nucleotide changes in individual mutants are indicated in red. (*b*) Alternative splicing of *IFT121* between exons 20–23. (i) Representation of multiple *IFT121* transcripts amplified within the region. (ii) RT-PCR products of *IFT121*. (*c*) RT-PCR of *FRA10* across the gene. Amplification of the ribosomal protein gene *CRY1* serves as a loading control.

In a mutant screen for aflagellate mutants, we isolated a new mutant strain, db35, that fails to assemble flagella (figure 3, *ift121-2*). Whole-genome sequencing indicates that it contains a 5′ splice site (donor) mutation, GT to AT, in the intron between exons 21 and 22 of *IFT121* (table 1, figure 4*a*). This mutant also contained a second mutation in a gene that is unlinked to *IFT121* (electronic supplementary material, table S2). We designed PCR based assays to detect both SNPs (electronic supplementary material, table S1) and selected a progeny (db35-1) that contains only the *IFT121* SNP to study further. A backcross of db35-1 showed cosegregation of the *IFT121* SNP and the aflagellate mutant phenotype (*n* = 20). Thus, this SNP is tightly linked to the mutant phenotype.

By RT-PCR, a single band from *IFT121* exons 20–23 is amplified in wild-type cells (figure 4*b*, band A), while two bands are amplified in the db35-1 mutant (figure 4*b*, *ift121-2*). Sanger sequencing indicates the larger band (band B) contains intron 21 (electronic supplementary material, figure S4A) and the smaller band (band C) contains a truncated exon 21, in addition to exons 20, 22 and 23. This transcript is generated by adoption of an alternative splice donor site, which is 31 nucleotides upstream of the original site, within exon 21 (electronic supplementary material, figure S4B).

To provide further evidence that the splice site mutation in *IFT121* is the causative mutation in this aflagellate mutant, we performed UV mutagenesis on the db35-1 mutant and identified revertants of the aflagellate phenotype of *ift121*. Two new strains (rev26 and rev28) show restored flagellar assembly (figure 3) and produce no aflagellate progeny when backcrossed to a wild-type strain. Sanger sequencing revealed that both mutants carry changes in the *IFT121* gene. In rev26, there is a synonymous mutation of K916 (AAG to AAA) in exon 21 that is 7 nucleotides upstream of the original mutation (figure 4*a*, electronic supplementary material, figure S4C). RT-PCR of *IFT121* in rev26 (figure 4*b*) reveals correct splicing of exons 20–23 with the silent mutation (Band A′), in addition to the alternatively spliced *IFT121* transcripts found in *ift121-2* (Bands B and C). Rev28 has a donor site mutation (GT to AT) at the beginning of intron 22 (figure 4*a*, electronic supplementary material, figure S4D), at position 2 411 221 on chromosome 11. It generates a complex set of *IFT121* transcripts (figure 4*b*, electronic supplementary material, figure S4D) that includes band D (exon 20, deletion of 31 nucleotides from exon 21, exon 22, intron 22 and exon 23) and band C′ (deletion of 31 nucleotides from exon 21, exon 22, inclusion of 7 nucleotides of intron 22 and exon 23). The resulting band C’ now restores an in-frame *IFT121* transcript. This transcript replaces 32 amino acids (aa 909–940) from the IFT121 protein sequence (black box, electronic supplementary material, figure S5) with 24 different amino acids. This change affects a few amino acids that are conserved across different species (electronic supplementary material, figure S5). The secondary structure predicted by YASPIN [41] indicates the C-terminus half of the IFT121 protein contains several predicted α-helices (electronic supplementary material, figure S5, magenta blocks). Three small α-helices (aa 899–915; aa 919–929; aa 932–950) are predicted within the region of replacement. Instead, now one large α-helix (aa 900–942) is predicted in the rev28 strain. Therefore, even though amino acid composition is changed around this region, the preserved secondary structure appears to be sufficient to restore flagellar assembly and motility in the *ift121-2 rev28* strain. Based on the swimming phenotype and *IFT121* splicing events found in these two intragenic revertants, we conclude the splice donor site mutation of *IFT121* is the causative mutation and renamed the db35-1 strain *ift121-2*.

### 3.5. A frameshift mutation of *FRA10* suppresses the *ift121-2* mutation

In addition to the intragenic revertants, we isolated two extragenic suppressors of the *ift121-2* mutant. One suppressor, sup15, splices *IFT121* correctly across exons 20–23 (figure 4*b*, *ift121-2; fra10*). Thus, it is likely to be a suppressor that affects splicing. The other suppressor, sup25, retains alternative *IFT121* splicing fragments. It is likely to be a suppressor that affects flagellar motility/assembly or mRNA stability but not splicing. Whole-genome sequencing of sup15 (electronic supplementary material, table S1) indicates a single nucleotide deletion (TG to T, table 1), which leads to a frameshift, in the *Cre07.g336250* gene. The sup15 strain was backcrossed to wild-type. Fourteen meiotic progeny that contain the *ift121-2* mutation but show wild-type flagellar assembly cosegregate with the single nucleotide deletion in *Cre07.g336250*. This gene encodes a protein that shares 54% identity and 69% similarity (3 × 10^−55^) to the human folate-sensitive fragile site protein FRA10AC1 (electronic supplementary material, figure S6). We named it *FRA10* in *Chlamydomonas*.

We transformed the *ift121-2*; *fra10* double mutant with a 3.1 kb DNA fragment that includes full-length wild-type *FRA10* gene and approximately 0.9 kb upstream DNA. It is expected that rescue of the *fra10* mutant results in aflagellate cells as observed in *ift121-2*. We obtained 10 aflagellate transformants. Five of them contain the wild-type *FRA10* gene (*ift121-2; fra10; FRA10-TG*) while the other five may generate the aflagellate phenotype through random insertion [51]. The *IFT121* transcript profiles in all five *FRA10-TG* transformants, obtained from four independent transformations, were analysed. The transcript products found in all *ift121-2; fra10; FRA10-TG* transformants and in *ift121-2* are similar (figure 4*b* and electronic supplementary material, figure S8). Therefore, the frameshift mutation of *FRA10* acts as a suppressor to restore the wild-type splicing pattern in *ift121-2.* The abundance of *FRA10* transcripts in both *ift121-2*; *fra10* and *ift121-2; fra10; FRA10-TG* is similar to the levels in wild-type (CC-125) (figure 4*c*). Given the single-nucleotide deletion, which is predicted to cause a frameshift, is found in the last exon (exon 4) of the gene, the mutant transcript is unlikely to be subjected to NMD [53].

### 3.6. *DGR14* and *FRA10* mutations can suppress both splice donor and acceptor site mutations

The *dgr14* mutations suppress the splice acceptor site in *fla9* and the *fra10* mutation suppresses the splice donor site in *ift121-2*. Since both DGR14 and FRA10 were identified as spliceosomal C complex proteins [13,14] and they show protein–protein interaction [2], we asked whether mutations in these two spliceosomal proteins suppress both splice donor and acceptor site mutations. Flagellar assembly is restored in both *fla9; fra10* and *ift121-2; dgr14-1* strains (figure 3). Correspondingly, the wild-type *IFT81* transcript is restored in the *fla9*; *fra10* double mutant (figure 2*d*) and the wild-type *IFT121* transcript is restored in the *ift121-2*; *dgr14-1* mutant (figure 4*b*). Cells are aflagellate in *fla9*; *fra10*; *FRA1-TG* and in *ift121-2*; *dgr14-1*; *DGR14-TG* strains (figure 3). We conclude that a mutation in either *DGR14* or *FRA10* is sufficient to suppress both splice donor and acceptor site mutations in these *IFT* genes.

### 3.7. Nonsense mutations in the *SMG1* gene stabilize the misspliced transcripts in the *ift* mutants

**Figure 5.**
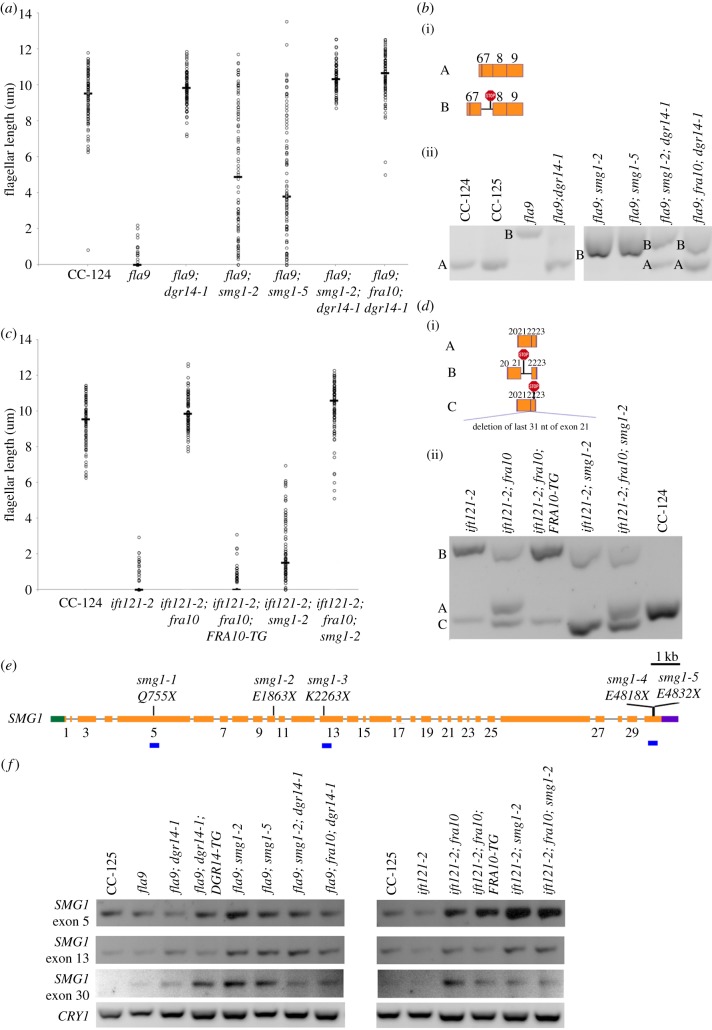
Nonsense mutations in *smg1* affect flagellar assembly in splice site *ift* mutants. (*a*) Distribution of flagellar length in wild-type (CC-124) and various *fla9* mutants. Lengths of 100 flagella (represented by open circles) from 50 cells were measured in each strain. Horizontal bar represents the median flagellar length in each strain. (*b*) Alternative splicing of *IFT81* between exons 6–9. (i) Representation of wild-type and intron retention transcripts. The red stop sign represents premature termination codon. (ii) RT-PCR products of *IFT81* amplified from the same cells used in (*a*). (*c*) Distribution of flagellar length in wild-type (CC-124) and various *ift121-2* mutants. Lengths of 100 flagella (represented by open circles) from 50 cells were measured in each strain. Horizontal bar represents the median flagellar length in each strain. (*d*) Alternative splicing of *IFT121* between exons 20 to 23. (i) Representation of multiple *IFT121* transcripts amplified within the region. (ii) RT-PCR products of *IFT121* amplified from the same cells used in (*c*). (*e*) Gene structure of *SMG1* and positions of multiple nonsense mutations. Green box, 5′ UTR; orange boxes, exons; black solid lines, introns; purple box, 3′ UTR; vertical black lines, positions of *smg1* nonsense mutations. The positions of individual mutations in the SMG1 protein are indicated. Numbers of exons are indicated below the orange boxes. Owing to limitation of space, only odd numbers are included. The blue horizontal bars indicate regions amplified by RT-PCR in (*f*). (*f*) RT-PCR products of *SMG1* in multiple exons. Amplification of the ribosomal protein gene *CRY1* serves as a loading control.

In an independent screen for suppressors of a paralyzed flagella mutant, we identified five nonsense mutants in the *SMG1* gene (*Cre13.g572050*) (table 1; figure 5*e*) by whole-genome sequencing. *Chlamydomonas* SMG1 protein shares 32% identity and 46% similarity to its human homologue (1 × 10^−110^) (electronic supplementary material, figure S7). Since one of the important roles of NMD is to remove transcripts harbouring a premature terminated codon (PTC), we asked whether the *smg1* mutations affect the misspliced *IFT81* transcripts in *fla9* and the misspliced *IFT121* transcripts in *ift121-2.*

We generated three double mutant strains (*fla9*; *smg1-2*, *fla9*; *smg1-5* and *ift121-2*; *smg1-2*) and two triple mutants (*fla9; dgr14-1; smg1-2* and *ift121-2; fra10; smg1-2*). While both *fla9* and *ift121-2* single mutant strains have very short or no flagella, both mutants show various flagellar lengths in the *smg1* background (figure 5*a,c*). However, these cells display no motility. The triple mutant cells, similar to the *fla9*; *dgr14-1* or *ift121*; *fra10* double mutants, are motile and have normal flagellar length.

Semi-quantitative RT-PCR of *IFT81* (figure 5*b*) followed by Sanger sequencing revealed that in the *fla9*; *smg1-2* double mutant, the abundance of the intron inclusion transcript (band B), which bears a PTC, is approximately fivefold greater than that in the *fla9* mutant. In the *fla9*; *smg1-5* double mutant, the abundance is approximately fourfold greater. No wild-type transcript (band A) is observed in either double mutant. This suggests that *IFT81* splicing is not altered in the *smg1* mutant background. In the triple mutants *fla9; dgr14-1; smg1-2* and *fla9*; *dgr14-1*; *fra10*, both the wild-type and intron inclusion transcripts are detected. The abundance of these transcripts is not significantly increased.

In the *ift121-2*; *smg1-2* double mutant, the abundance of the truncated transcript (band C), which contains a PTC, increases approximately ninefold (figure 5*d*). Interestingly, no accumulation of the intron inclusion transcript (band B), which also harbours a PTC, is observed in the double mutant. A close examination of the transcript sequence reveals that it contains four in-frame AUG codons within 200 nucleotides downstream of the PTC, a widespread mechanism used by human genes to escape NMD surveillance [54]. No wild-type *IFT121* transcript (band A) is observed in the double mutant. In the *ift121-2*; *fra10*; *smg1-2* triple mutant, we detected all three transcripts and there is about fourfold accumulation of the truncated transcript (band C) but not in the intron inclusion transcript (band B).

The *smg1-2* mutation does not affect the abundance of the mutant *fra10* transcript in the *ift121-2*; *fra10*; *smg1-2* mutant (figure 4*c*). This is consistent with our hypothesis that the frameshift in the last exon of the *FRA10* transcript is not subjected to NMD. In contrast, the nonsense *SMG1* mutant transcripts accumulate in both the *smg1-2* and *smg1-5* mutant strains (figure 5*f*). It indicates that the NMD pathway is likely to be compromised in the *smg1* mutants.

## 4. Discussion

### 4.1. Misregulation of RNA splicing via mutations in *cis*-acting RNA sequences

RNA splicing is necessary to produce mature RNA for almost all genes in vertebrates. Misregulation of RNA splicing, by both *cis-*acting RNA sequences and *trans*-acting RNA splicing factors, are linked to cancers and other human diseases [3,55].

In this study, we report mutations in the splice sites of two *Chlamydomonas IFT* genes that lead to aberrant splicing of their transcripts and defects in flagellar assembly. The isolation of two intragenic revertants of *ift121-2* shows novel ways that cells can rescue a splice site defect. In *ift121-2*, the mutation in the donor splice site in intron 21 leads to an alternative donor site 31 nucleotides upstream (electronic supplementary material, figure S4A). Exonic splicing enhancers (ESEs) are short oliognucleotide sequences in exons around the splice sites that bind to splicing factors and facilitate splicing [56]. It is estimated that approximately 4% of synonymous changes are deleterious to splicing by affecting ESE sequences [57]. We used the RESCUE program for human ESE prediction [56], and it reveals nine ESEs within 12 nucleotides upstream of the canonical splice donor site of intron 21. In the *ift121-2 rev26* mutant, a synonymous change that is 7 nucleotides upstream of the original mutation partially restores wild-type splicing (electronic supplementary material, figure S4B). The single nucleotide change in *rev26* is predicted to change the sequences of eight ESEs and to add a new ESE. These ESEs may have higher affinity for splicing factors around the noncanonical splice site (AT in *ift121-2*) and this recruitment could facilitate splicing.

A genome-wide analysis of alternative spliced transcripts in *Chlamydomonas* indicated that both constitutive splicing and alternative splicing events use GT as the consensus splice donor site [58]. However, in the *ift121-2 rev28* mutant, the splicing machinery opts for a noncanonical splice donor site (GC). It is unclear why this site is chosen, but it results in an in-frame reading frame. While this choice changes the primary protein sequence, it is unlikely to change the secondary structure based on structure predictions [41].

### 4.2. Correction of RNA splicing mistakes via mutations in spliceosomal proteins

Both DGCR14 and FRA10AC1 were identified in proteomic studies of human spliceosomes but are absent in yeast spliceosomes [2]. While yeast and human spliceosomes share common core structures and proteins, human spliceosomes contain more spliceosomal proteins that play regulatory roles [59]. Analysis of yeast postcatalytic spliceosome structure [60–62] revealed formation of non-Watson–Crick base pairings between G (+1) of the 5′ splice site and G (−1) of the 3′ splice site, and between A (−2) of the 3′ splice site and the conserved adenine at the branch point, which is linked to G (+1) of the 5′ splice site. While the structure of human postcatalytic spliceosome has not been revealed yet, we expect it uses similar base pairing mechanisms but contains more spliceosomal proteins that may include DGCR14 and FRA10AC1. In our study, the splice site mutations disrupt G (+1) of the 5′ splice site and A (−2) of the 3′ splice site. These mutations in *dgr14* and *fra10* mutants show increased wild-type splicing. Therefore, DGR14 and FRA10 are likely to be involved in facilitating recognition/interaction among G (+1), A (−2) and A at the branch point. However, it is unclear whether the involvement is direct or indirect and it will require additional assays to address their functions.

We observed no obvious defects in viability, mating efficiency and motility in the single mutants of *dgr14* and *fra10* or the *dgr14-1*; *fra10* double mutant. This is consistent with the observation that deletion of Bis1, the DGCR14 homologue found in fission yeast, does not affect viability during exponential growth [7]. We performed transcriptome analysis of *dgr14*, *fra10* and the double mutant but find very few changes in RNA splicing and abundance (M Pandey, G Stormo and SK Dutcher 2018, unpublished work). Similarly, no phenotypic or splicing defect has been report in the *ess-2* mutant in *C. elegans* [8].

**Figure 6.**
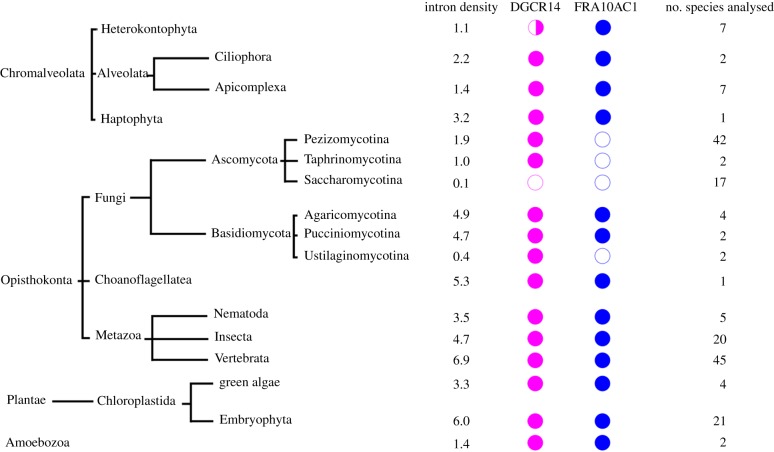
Distribution of orthologues of DGCR14 and FRA10AC1 in multiple eukaryotic kingdoms. The median intron density (per 1 kb coding region) in each class is calculated from Rogozin *et al.* [43]. Open circles, protein absent from the class; half closed circles, protein absent from some species within the class; closed circles, protein present in the class. The lengths of branches do not represent evolutionary distance. The numbers of species analysed in each class are indicated on the right.

Given that both DGCR14 and FRA10AC1 are not core splicing proteins [2], we asked whether these two proteins are present in different species across multiple eukaryotic kingdoms (figure 6). The presence/absence of orthologues of each protein in individual species was extracted from the EggNOG database [42] and NCBI BLAST. In *Saccharomyces cerevisiae*, approximately 5% of genes contain introns and its spliceosome contains fewer than 100 proteins [63,64]. The intron density per 1 kb of coding sequence is approximately 0.1 [43]. In contrast, approximately 43% of *Schizosaccharomyces pombe* genes contain introns and its intron density is ten-fold higher [43,64]. The DGCR14 homologue Bis1, which is proposed to be a stress response protein [7], is present in four different species of the fission yeasts [64], while both DGCR14 and FRA10AC1 homologues are absent in the Saccharomycetes lineage (*n* = 17). In addition to Saccharomycetes, DGCR14 is absent in the oomycetes *Phytophthora ramorum* (Pram) and *Phytophthora capsici* (Pcap). However, the genome of *Phytophthora sojae* contains DGCR14. Therefore, the absence of DGCR14 in Pram and Pcap may arise from incomplete genome assembly. Even though DGCR14 is not essential to RNA splicing, it is present in most species analysed (figure 6). FRA10AC1 is absent in Ascomycota, a subgroup of fungi. It is also absent in Ustilaginomycotina, which have very low intron density (approx. 0.4) when compared to other organisms in Basidiomycota [43]. The absence of FRA10AC1 correlates with low intron density and we suggest that these two observations may be related. An exception is observed in the unicellular green algae *Ostreococcus*, in which the intron density is low (approx. 0.6) but both FRA10AC1 and DGCR14 are present. In general, higher intron density (greater than 3) may require additional noncore spliceosomal proteins to recognize intron boundaries and promote splicing, therefore the presence of both proteins is observed in these species. In *Chlamydomonas*, 88% of the genes contain introns and an intron density of approximately 6.3, which is similar to the vertebrates with an intron density of 6.9 [43,58].

### 4.3. The *ift* mutants provide new insights about intraflagellar transport

Mutations of *IFT81* in humans lead to multiple symptoms that include polydactyly, nephronophthisis [65], asphyxiating thoracic dystrophy, short rib polydactyly [66] and retinal dystrophy [65,67]. In the *fla9* mutant strain reported here, the most prevalent transcript shows retention of *IFT81* intron 7 and produces no protein that is detected by the monoclonal anti-IFT81 antibody [47], which recognizes the C-terminus of IFT81 (D Cole 2014, personal communication, figure 2*c* and electronic supplementary material, figure S2). In the *ift81-1* mutant strain, which was generated by an insertion in exon 7 of *IFT81*, no IFT81 protein was detected by immunoblot with the same antibody [23]. We propose that the *fla9* mutant transcript produces a truncated IFT81 protein that contains the first approximately 270 amino acids, not detected by the antibody. It has been reported that the N-termini of IFT81 and IFT74 are crucial to flagellar assembly and may dimerize to form a binding module for tubulin [23,68]. In the *fla9*; *smg1* double mutant, accumulation of this transcript produces enough truncated IFT81 proteins to interact with the N-terminus of IFT74 and to allow variable flagellar assembly (figure 5*a*). It is worth noting that the flagella remain immotile in *fla9*; *smg1* cells, which suggests the C-terminus of IFT81 is required for cargo needed for flagellar motility.

Mutations in *IFT121*/*WDR35* result in cranioectodermal dysplasia [69–73], short rib polydactyly syndrome [74], Ellis–van Creveld syndrome [75] and respiratory dysfunction [72]. Studies using truncation mutants indicated the N-terminus of IFT121 (aa 1–640) is important for interactions with IFT122, Arl13b and INPP5E while the C-terminus (aa 641–1181) is important for its ciliary localization [76]. In the *ift121-2*; *smg1-2* mutant, the accumulated misspliced transcript (Band C in figure 5*d*) is expected to encode a truncated IFT121 protein that contains the first 908 amino acids with an additional 56 novel amino acids. We propose that accumulation of this truncated IFT121 protein is responsible for flagellar assembly. An insertional mutant of *IFT121* (*ift121-1*) has been previously reported. The exact location of gene disruption is unknown but it is within the last one-third of the gene [77]. Similar to our *ift121-2* mutant, the *ift121-1* mutant is aflagellate and it is unclear whether *ift121-1* can assemble flagella in a *smg1* background.

Hypoxia has been reported to partially restore flagellar assembly in truncated IFT46 and IFT74 mutants [78,79]. Removal of the first 196 amino acids from IFT74 in the *ift74-1* mutant leads to a slight accumulation of the truncated protein and the mutant cells assemble immotile flagella when cells are not aerated [79]. When stressed, a truncated IFT46 protein, which lacks the N-terminal 100 amino acids, accumulates and the mutant cells assemble flagella with various lengths [78]. Our study on *fla9*; *smg1* and *ift121-1*; *smg1* mutants provides additional evidence that over-accumulation of truncated IFT proteins can partially rescue flagellar assembly defects. To further understand the detailed mechanism, over-expression of truncated constructs of *IFT81* and *IFT121*, and their interactions with other IFT proteins, will be necessary.

## Supplementary Material

Supplemental Figures 1 - 5

## Data accessibility

The sequence data for the strains in the electronic supplementary material, table S2 are deposited at BioProject ID PRJNA407207 at the National Center for Bioinformatics Information (NCBI). All other data are presented in the electronic supplementary material, tables and figures.
