# Supplementary material for: Identifying RNA splicing factors using IFT genes in Chlamydomonas reinhardtii
Source: Open Biol. 2018 Mar 7;8(3):170211. doi: 10.1098/rsob.170211 (PMC5881031; doi:10.1098/rsob.170211)
Supplement: Supplemental Figures 1 - 5 [file rsob170211supp1.pdf]

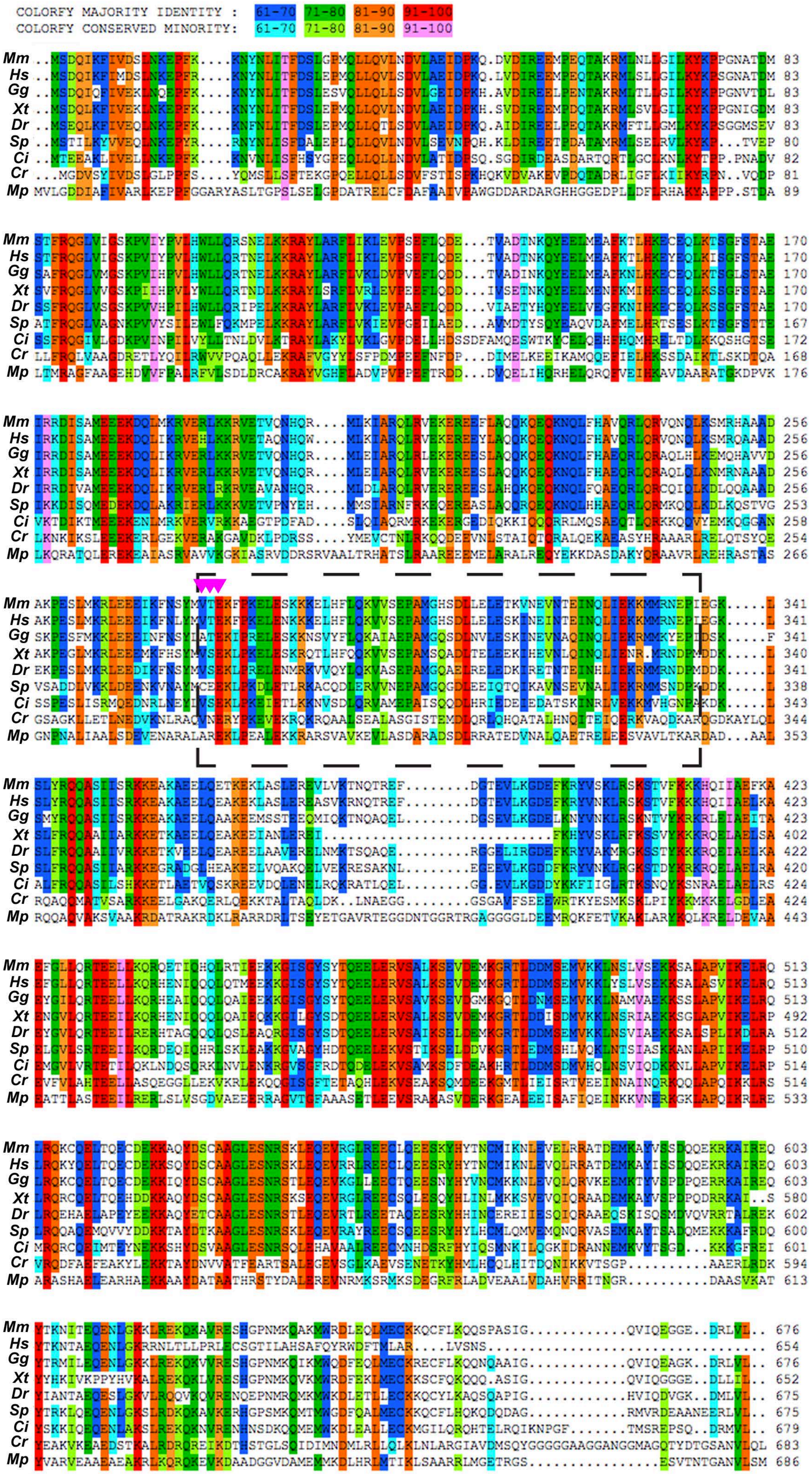
COLORFY MAJORITY IDENTITY : 61-70 71-80 81-90 91-100
COLORFY CONSERVED MINORITY: 61-70 71-80 81-90 91-100
Mm
Hs
Gg
Xt
Dr
Sp
Ci
Cr
Mp

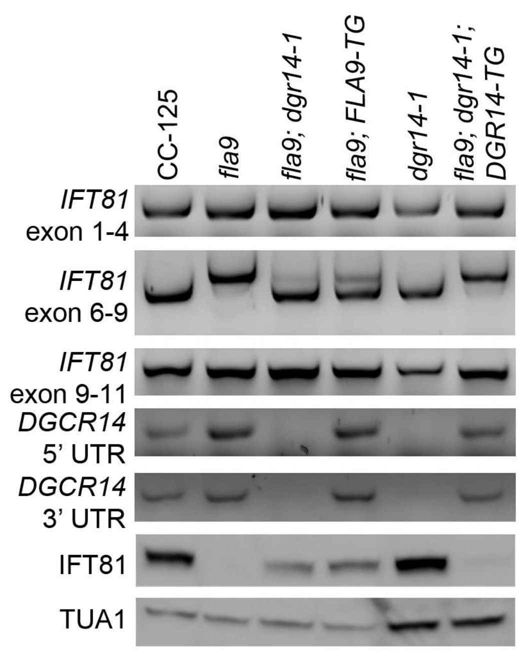
CC-125
fla9
fla9; dgr14-1
fla9; FLA9-TG
dgr14-1
fla9; dgr14-1; DGR14-TG
IFT81 exon 1-4
IFT81 exon 6-9
IFT81 exon 9-11
DGCR14 5' UTR
DGCR14 3' UTR
IFT81
TUA1

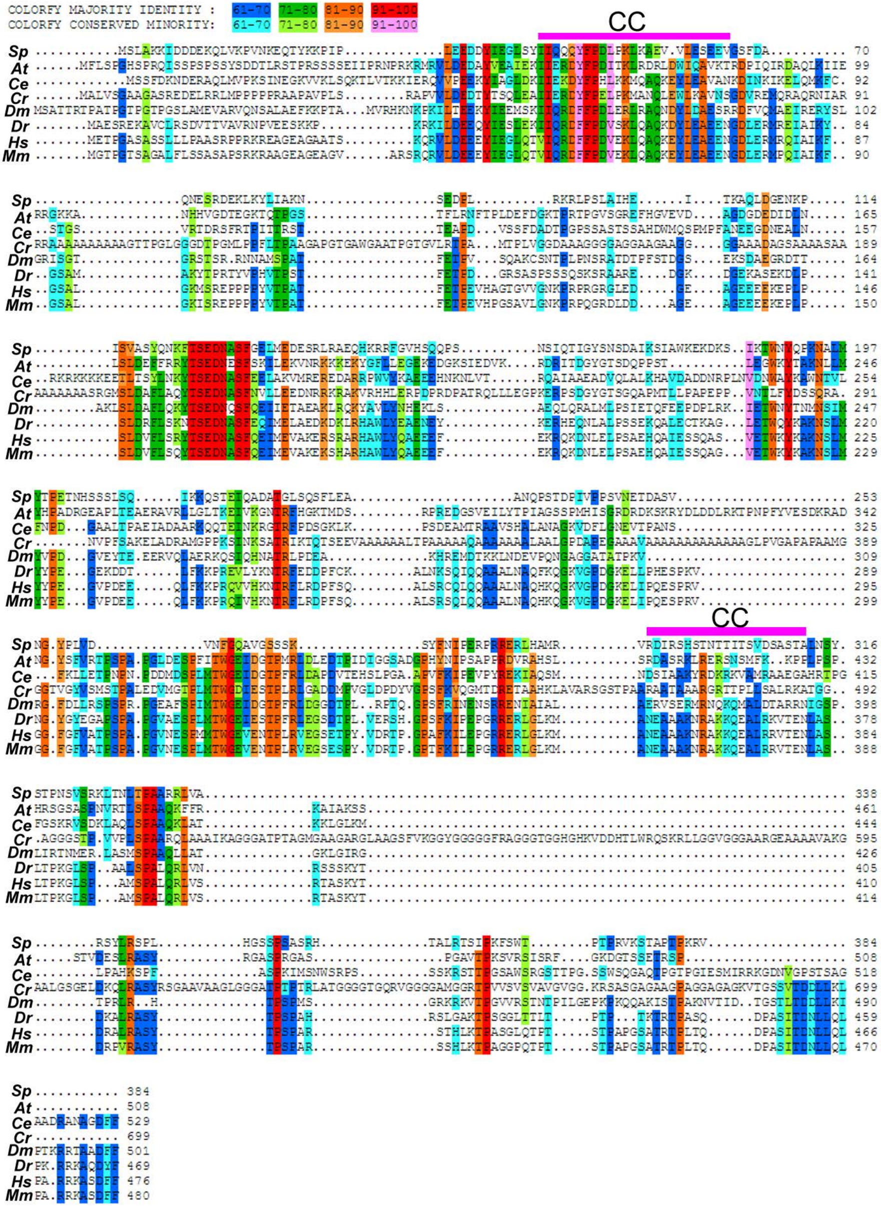
COLORFY MAJORITY IDENTITY : 61-70 71-80 81-90 91-100
COLORFY CONSERVED MINORITY: 61-70 71-80 81-90 91-100
CC
CC
Sp
At
Ce
Cr
Dm
Dr
Hs
Mm

A. *ift121-2* intron retention transcript

TTCCAGGT......AG**AGCGTG......CCAAGTACGCCAACCACCTCCTGGAGAAGGAGAAG**GTAGGCGGGGC

change to A

GGGGCATTGCCACGGGTTTAGGTAGCTTGAGCGGCAGCAGGACTTCCATGCAGTTTGCGGGGCGTGTATGTGAA

PTC generated by inclusion of intron 21

TGGTCTTAATTTGATTGCCATTATCGAGTCGTTGTATTGATCACGTGTGTATCCGGGTGCAG**GTCATGGACGCC**

putative start codon to resume translation

ATCGAGCTGTACCGCAAGGCCAACCACTCCATGGAGGCGGCTCGGCTCCTGCAGGTGGGACGCAAGTGGGCTGG
ATCATGGGGGGCAGAGAGCCGGACCGCTGTGTTTCAGCCACACCCTAGGAGCCATTGCGCCTCTGACACGCACC
TGCGGCCCGGGCCCGTGCACCCCCACAG**GACCTGGCAAAGAAGTGTGCGGACCAGAAGGTGCACCCGCTACGCG**
TTAAGAAGCTGTACGTGCTGGCGGCGCTGGAGATTGAGAAATTCAAGAAGCGCACGCTAGACATGGCAGGGCCG
GACAAGACCGCCGCCACGCTGCTGGGCACCGGCGGCACCG

B. *ift121-2* exon truncation transcript

TTCCAGGT......AG**AGCGTG......CCAAGTACGCCAACCACCTCCTGGAGAAGGAGAAG**GTAGGCGGGGC

new donor splice site   change to A

GGGGCATTGCCACGGGTTTAGGTAGCTTGAGCGGCAGCAGGACTTCCATGCAGTTTGCGGGGCGTGTATGTGAA
TGGTCTTAATTTGATTGCCATTATCGAGTCGTTGTATTGATCACGTGTGTATCCGGGTGCAG**GTCATGGACGCC**
ATCGAGCTGTACCGCAAGGCCAACCACTCCATGGAGGCGGCTCGGCTCCTGCAGGTGGGACGCAAGTGGGCTGG
ATCATGGGGGGCAGAGAGCCGGACCGCTGTGTTTCAGCCACACCCTAGGAGCCATTGCGCCTCTGACACGCACC
TGCGGCCCGGGCCCGTGCACCCCCACAG**GACCTGGCAAAGAAGTGTGCGGACCAGAAGGTGCACCCGCTACGCG**
TTAAGAAGCTGTACGTGCTGGCGGCGCTGGAGATTGAGAAATTCAAGAAGCGCACGCTAGACATGGCAGGGCCG

PTC generated by deletion of 31 nt of exon 21

GACAAGACCGCCGCCACGCTGCTGGGCACCGGCGGCACCG

C. *ift121-2 rev26*

TTCCAGGT......AG**AGCGTG......CCAAGTACGCCAACCACCTCCTGGAGAAGGAGAAG**GTAGGCGGGGC

change to A   change to A

GGGGCATTGCCACGGGTTTAGGTAGCTTGAGCGGCAGCAGGACTTCCATGCAGTTTGCGGGGCGTGTATGTGAA
TGGTCTTAATTTGATTGCCATTATCGAGTCGTTGTATTGATCACGTGTGTATCCGGGTGCAG**GTCATGGACGCC**
ATCGAGCTGTACCGCAAGGCCAACCACTCCATGGAGGCGGCTCGGCTCCTGCAGGTGGGACGCAAGTGGGCTGG
ATCATGGGGGGCAGAGAGCCGGACCGCTGTGTTTCAGCCACACCCTAGGAGCCATTGCGCCTCTGACACGCACC
TGCGGCCCGGGCCCGTGCACCCCCACAG**GACCTGGCAAAGAAGTGTGCGGACCAGAAGGTGCACCCGCTACGCG**
TTAAGAAGCTGTACGTGCTGGCGGCGCTGGAGATTGAGAAATTCAAGAAGCGCACGCTAGACATGGCAGGGCCG
GACAAGACCGCCGCCACGCTGCTGGGCACCGGCGGCACCG

D. *ift121-2 rev28*

TTCCAGGT......AG**AGCGTG......CCAAGTACGCCAACCACCTCCTGGAGAAGGAGAAG**GTAGGCGGGGC

new donor splice site   change to A

GGGGCATTGCCACGGGTTTAGGTAGCTTGAGCGGCAGCAGGACTTCCATGCAGTTTGCGGGGCGTGTATGTGAA
TGGTCTTAATTTGATTGCCATTATCGAGTCGTTGTATTGATCACGTGTGTATCCGGGTGCAG**GTCATGGACGCC**
ATCGAGCTGTACCGCAAGGCCAACCACTCCATGGAGGCGGCTCGGCTCCTGCAGGTGGGACGCAAGTGGGCTGG

change to A   new donor splice site

ATCATGGGGGGCAGAGAGCCGGACCGCTGTGTTTCAGCCACACCCTAGGAGCCATTGCGCCTCTGACACGCACC
TGCGGCCCGGGCCCGTGCACCCCCACAG**GACCTGGCAAAGAAGTGTGCGGACCAGAAGGTGCACCCGCTACGCG**
TTAAGAAGCTGTACGTGCTGGCGGCGCTGGAGATTGAGAAATTCAAGAAGCGCACGCTAGACATGGCAGGGCCG
GACAAGACCGCCGCCACGCTGCTGGGCACCGGCGGCACCG

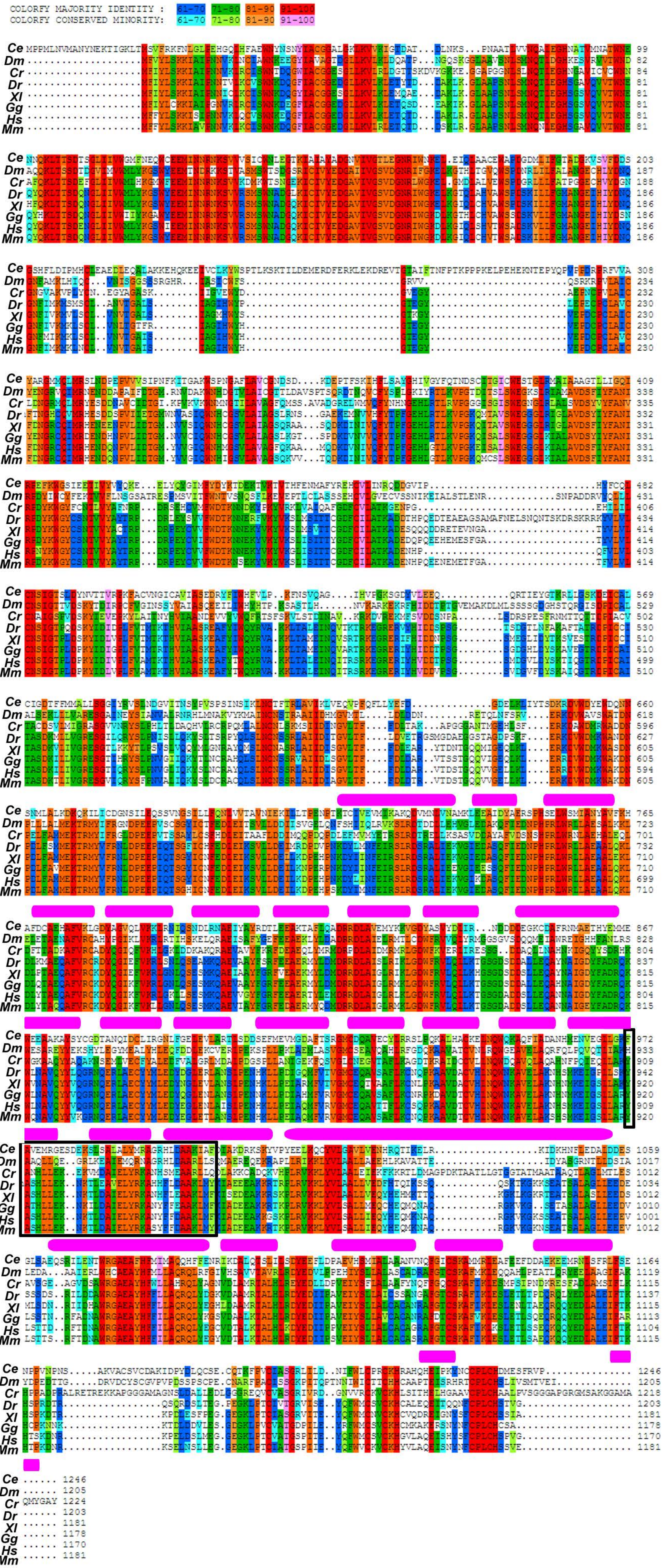
COLORFY MAJORITY IDENTITY : 61-70 71-80 81-90 91-100
COLORFY CONSERVED MINORITY: 61-70 71-80 81-90 91-100

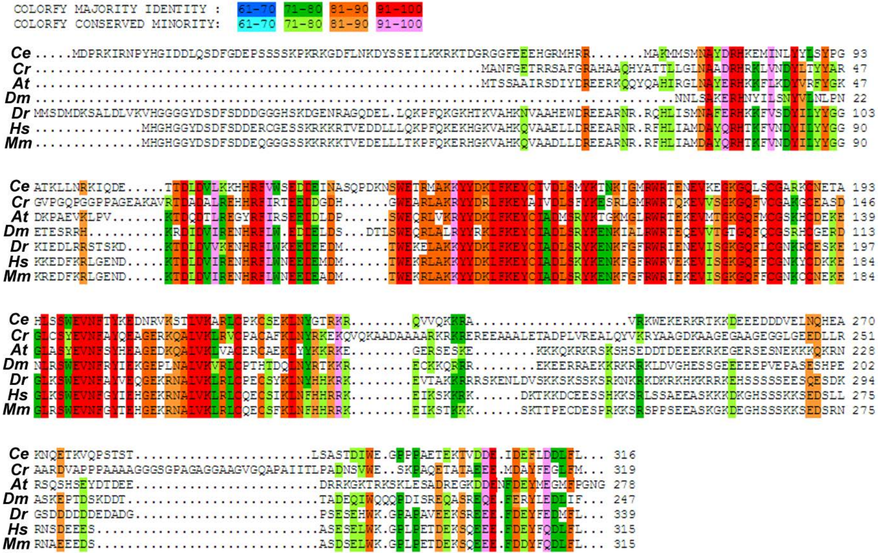
COLORFY MAJORITY IDENTITY : 61-70 71-80 81-90 91-100
COLORFY CONSERVED MINORITY: 61-70 71-80 81-90 91-100
Ce
Cr
At
Dm
Dr
Hs
Mm

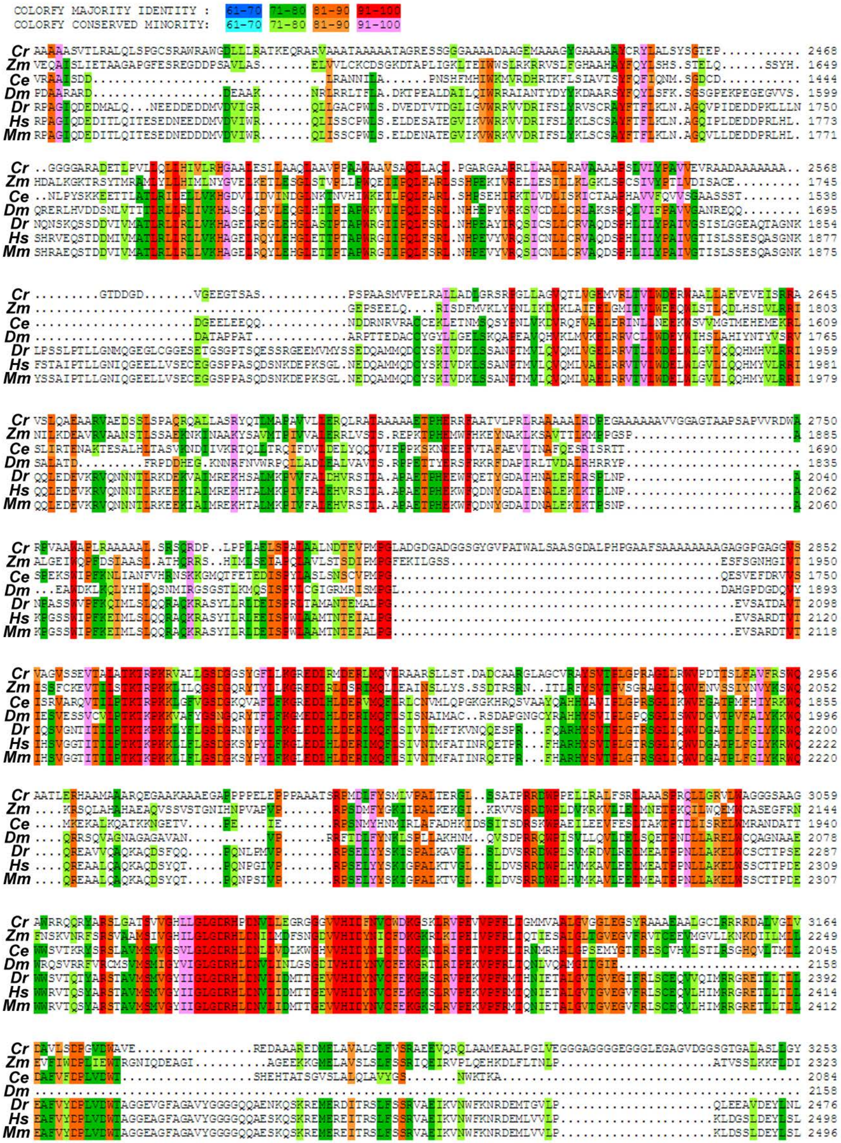
COLORFY MAJORITY IDENTITY : 61-70 71-80 81-90 91-100
COLORFY CONSERVED MINORITY: 61-70 71-80 81-90 91-100

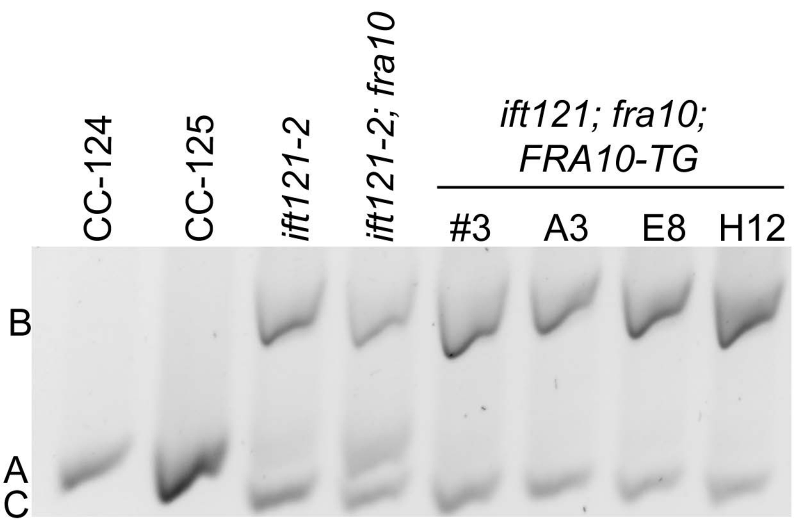
CC-124
CC-125
ift121-2
ift121-2; fra10
ift121; fra10; FRA10-TG
#3
A3
E8
H12
B
A
C

Supplemental Figure 1. Protein sequence alignment of IFT81. Sequence similarity percentage is displayed by colors shown above the alignment. The position of exon 8, which is skipped in some of the *fla9* transcripts, is indicated in the dashed black box. The three amino acids ($V_{275}N_{276}E_{277}$) that are deleted in some of the *fla9* transcripts, are indicated by reversed triangles in magenta. Abbreviations of species and NCBI accession numbers of individual proteins used in alignment are the following: *Ci, Ciona intestinalis*, XP_002126367.1; *Cr, Chlamydomonas reinhardtii,* XP_001697224.1; *Dr, Danio rerio*, AAI63882.1; *Gg, Gallus gallus*, NP_001239238.2; *Hs*, *Homo sapiens,* NP_001334876.1; *Mm, Mus musculus*, NP_034009.2; *Mp, Micromonas pusilla CCMP1545,* XP_003061494.1; *Sp, Strongylocentrotus purpuratus*, XP_011667377.1; *Xt, Xenopus tropicalis*, XP_004910569.1.

Supplemental Figure 2. The *IFT81* transcripts and the IFT81 protein at 32˚C. The cells were initially incubated at 21˚C for 2 hours. One-half of the cells was transferred to 32˚C and incubated for 4 hours while the other was maintained at 21˚C (Figure 2B and 2C). Isolation of both RNA (top four panels) and proteins (bottom two panels) from samples at both temperatures were performed at the end of 6 hours since first incubation.

Supplemental Figure 3. Protein sequence alignment of DGR14. Sequence similarity percentage is displayed by colors shown above the alignment. Two predicted coiled coil domains (CC) are labeled. The second CC domain is predicted in the human protein but not in the *Chlamydomonas* homolog. Abbreviations of species and NCBI accession numbers (unless indicated otherwise) of individual proteins used in alignment are the following: *At, Arabidopsis thaliana*, NP_187436.1; *Ce, Caenorhabditis elegans*,

NP_001022580.1; *Cr, Chlamydomonas reinhardtii,* Cre11.g482050 (*Chlamydomonas* Phytozome genome assembly v5.5); *Dm, Drosophila melanogaster*, NP_572480.1; *Dr, Danio rerio*, NP_001082799.2; *Hs*, *Homo sapiens,* NP_073210.1; *Mm, Mus musculus,* AAC40077.1; *Sp, Schizosaccharomyces pombe*, NP_587842.1.

Supplemental Figure 4. Nucleotide sequences and splicing details of *IFT121* in various *ift121* mutants. Exon sequences are indicated with bold letters and intron sequences are indicate with plain letters. Mutations and splicing effects are indicated with underlines. (A) The *ift121-2* mutant contains an intron retention transcript. The single nucleotide mutation in *ift121-2* is indicated in magenta. The premature termination codon (PTC) is highlighted in red. The putative start codon to resume translation is highlighted in green. (B) The *ift121-2* mutant also contains a transcript that deletes the last 31 nucleotides of exon 21. Both single nucleotide mutation and the new donor splice site are indicated in magenta. The 31 nucleotides that are removed due to the choice of the new donor site are indicated in the dashed blue box. (C) The *ift121-2 rev26* mutant carries a single nucleotide mutation (highlighted in orange) 7 nucleotides upstream of the original mutation (highlighted in magenta). No deletion of 31 nucleotides is detected in this strain. (D) The *ift121 rev28* mutant contains a single nucleotide mutation (highlighted in blue) in intron 22. A new donor site that is 7 nucleotides downstream of this change is indicated in blue. The skipped 31 nucleotides (same as in (B)) are indicated in the dashed blue box and the newly included 7 nucleotides are indicated in the solid blue box.

Supplemental Figure 5. Protein sequence alignment of IFT121. Sequence similarity percentage is displayed by colors shown above the alignment. Alpha helixes predicted

by YASPIN are indicated by magenta blocks above the sequences. The amino acids that are replaced in *ift121 rev28* are indicated in the black box. Abbreviations of species and NCBI accession numbers of individual proteins used in alignment are the following: *Ce, Caenorhabditis elegans*, NP_508940.4; *Cr, Chlamydomonas reinhardtii,* XP_001702021.1; *Dm, Drosophila melanogaster*, NP_647653.1; *Dr, Danio rerio*, NP_001139061.1; *Gg, Gallus gallus*, XP_419970.3; *Hs, Homo sapiens,* AAH36659.1; *Mm, Mus musculus*, AAH56925.1; *Xl, Xenopus laevis*, XP_018121446.1.

Supplemental Figure 6. Protein sequence alignment of FRA10. Sequence similarity percentage is displayed by colors shown above the alignment. Abbreviations of species and NCBI accession numbers (unless indicated otherwise) of individual proteins used in alignment are the following: *At, Arabidopsis thaliana*, NP_193239.5; *Ce, Caenorhabditis elegans*, NP_001129806.1; *Cr, Chlamydomonas reinhardtii,* Cre07.g336250 (*Chlamydomonas* Phytozome genome assembly v5.5); *Dm, Drosophila melanogaster*, AAM48345.1; *Dr, Danio rerio*, NP_001006006.2; *Hs, Homo sapiens,* NP_660289.2; *Mm, Mus musculus*, NP_001074544.1.

Supplemental Figure 7. Partial protein sequence alignment of SMG1 around the highly-conserved phosphatidylinositol 3-kinase-related kinase domain. Sequence similarity percentage is displayed by colors shown above the alignment. Abbreviations of species and NCBI accession numbers of individual proteins used in alignment are the following: *Ce, Caenorhabditis elegans*, NP_001021053.1; *Cr, Chlamydomonas reinhardtii,* XP_001693485.1; *Dm, Drosophila melanogaster*, CAD89223.1; *Dr, Danio rerio*, NP_001073513.2; *Hs, Homo sapiens,* NP_055907.3; *Mm, Mus musculus,* NP_001026984.1; *Zm, Zea mays*, XP_008665371.1.

Supplemental Figure 8. *IFT121* splicing in *ift121-2*; *fra10*; *FRA10-TG* transformants. Four independent *ift121-2; fra10; FRA10-TG* transformants (#3, A3, E8, H12) all replicated the alternative splicing pattern of *IFT121* between exons 20 to 23 as found in *ift121-2*. Transformant #3 was used for further study. The A, B, C bands are identical to those described in Figure 4B.

Supplemental Table 1: Primers used in this study.

Strain	Forward primer sequence	Reversed primer sequence	Annealing temperature	Enzyme	Fragments (bp, wild-type; mutant)
fla9/ift81-2	CCGGTATTGTCTTAGGGAGCTT	ACTTTGCGCTCCTGGATCT	53˚C	*Fsp*I	168+135; 303
ift121-2	GCACAACTTCCCGCAGAT	CACTTCTTTGCCAGGTCCTG	53˚C	*Mbo*II	419; 343+76
4c/*dgr14-1*	CTTCCAAGGGTACCCGTAAT	GCTGGAGAGAAGATGCAGAG	53˚C	n/a	115; no amp
dgr14-2	TGGCAGAGGGAATGATGGAA	GCCACCGCCAGTTTGTG	53˚C	n/a	202; no amp
sup15/*fra10-1*	CATCCCAGCCTGCCGTCTC	GCCGCCTCCTCACGCTCGCGCTTA	61˚C	n/a	132; no amp
smg1-1	ACGAGGTGGCTTGGATGG	CTGCCACTGTTGCTGT	53˚C	*Acu*I	89+61; 150
smg1-2	CTTGCCTACAACGCCTTCC	GCAGGCGCGCAATTCATAAT	53˚C	*Bsr*BI	159+83; 242
smg1-3	CTGCTGCCACACCTTCAG	CCTGCTGTTGCTGCCTATG	53˚C	*Alu*I	169; 113+56
smg1-4	AAGAGCACGAACAGGCACAG	CGGTGGTGCTGCACATGTT	53˚C	*Mse*I	145; 125+20
smg1-5	CGCTATCCCATCTACTGCCA	CTCACGGCCCTCCAGCTT	53˚C	*Mnl*I	100+57+18; 157+18

Primer	Sequence
IFT81 ex1-ex4 F	TGAGCTACATCGTGGACAGC
IFT81 ex1-ex4 R	ATCGGGGAAGCTCAGGTAGT
IFT81 ex6-ex9 F	GAGGAGGTCAACCTGTCCAC
IFT81 ex6-ex9 R	GCTTGCTCTTCATGCTCTCA
IFT81 ex9-ex11 F	TGAGAGCATGAAGAGCAAGC
IFT81 ex9-ex11 R	GATGGCGTTGTTGATCTCCT
IFT121 rt-1F	ACATCGGCGAGAAGTTCCAG

IFT121 rt-1R	CACTTCTTTGCCAGGTCCTG
FRA10-1F	GGCACTGCGTCTAGTTGGGG
FRA10-1R	CCTCAGCTGCAGGGACGAAG
DGR14 5'F	CTTCCAAGGGTACCCGTAAT
DGR14 5'R	GCTGGAGAGAAGATGCAGAG
DGR14 3'F	CTTACGTCGCAGTTGGCTAT
DGR14 3'R	CAGGCACGGAGCAGTACTAT
SMG1 ex5 F	ACGAGGTGGCTTGGATGG
SMG1 ex5 R	CTGCCACTGTTGCTGT
SMG1 ex13 F	CTGCTGCCACACCTTCAG
SMG1 ex13 R	CCTGCTGTTGCTGCCTATG
SMG1 ex30 F	CGCTATCCCATCTACTGCCA
SMG1 ex30 R	CTCACGGCCCTCCAGCTT

Supplemental Table 2. Summary of whole genome sequencing results

Strain	Total reads	Aligned reads	Coverage	% Aligned	# of Total changes	# of Unique changes	# of Putative changes	Gene
4c	193037602	186941282	17	96.8	81824	1193	0	*DGR14*
db35	43153902	37130207	34	86.0	42347	4873	23	*IFT121, RPG1*
ift121-2; fra10	57912006	54666636	50	94.4	66592	3449	5	*FRA10*
smg1-1	44901754	37928187	51	84.5	41270	5667	66	*SMG1*
smg1-2	46883800	40780679	55	87.0	43036	6431	49	*SMG1*
smg1-3	64887908	58420470	79	90.0	46260	6524	68	*SMG1*
smg1-4	42498864	36026593	49	84.8	42988	6145	52	*SMG1*
smg1-5	68079544	61463903	83	90.3	62894	7727	64	*SMG1*
